# Kinetics of Proton Transfer and String of Conformational Transformation for 4-Pyridone-3-carboxylic Acid Under External Electric Field

**DOI:** 10.3390/molecules30153115

**Published:** 2025-07-25

**Authors:** Ya-Wen Li, Rui-Zhi Feng, Xiao-Jiang Li, Ai-Chuan Liu, En-Lin Wang

**Affiliations:** 1 School of Earth and Environment, Anhui University of Science and Technology, Huainan 232001, China; 2 School of Safety and Emergency Management Engineering, Taiyuan University of Science and Technology, Taiyuan 030024, China; ruizhi_feng@163.com; 3 Shanxi North Xingan Chemical Industry Limited Company, Taiyuan 030008, China; xiaojianglee@sohu.com (X.-J.L.); liuaichuan1976_ty@126.com (A.-C.L.); 4 School of Materials Science and Engineering, Beijing University of Chemical Technology, Beijing 100029, China; 2022020365@buct.edu.cn

**Keywords:** 4-pyridone-3-carboxylic acid, double-proton cooperativity transfer, conformational transformation, kinetics, FTS

## Abstract

In order to explore the essence of the anticoccidiosis of anticoccidial drugs under bioelectric currents, the intermolecular double-proton transfer and conformational transformation of 4-pyridone-3-carboxylic acid were investigated by quantum chemistry calculations (at the M06-2X/6-311++G**, M06-2X/aug-cc-pVTZ and CCSD(T)/aug-cc-pVTZ levels) and finite temperature string (FTS) under external electric fields. The solvent effect of H_2_O on the double-proton transfer was evaluated by the integral equation formalism polarized continuum model. The results indicate that the influences of the external electric fields along the direction of the dipole moment on double-proton transfer are significant. The corresponding products are controlled by the direction of the external electric field. Due to the first-order Stark effect, some good linear relationships form between the changes of the structures, atoms in molecules (AIMs) results, surface electrostatic potentials, barriers of the transition state, and the external electric field strengths. From the gas to solvent phase, the barrier heights increased. The spatial order parameters (*ϕ*, *ψ*) of the conformational transformation could be quickly converged through the umbrella sampling and parameter averaging, and thus the free-energy landscape for the conformational transformation was obtained. Under the external electric field, there is competition between the double-proton transfer and conformational transformation. The external electric field greatly affects the cooperativity transfer, while it has little effect on the conformational transformation. This study is helpful in the selection and updating of anticoccidial drugs.

## 1. Introduction

By adjusting the strength and orientation of the external electric field, the barrier heights of the proton transfer can be reduced to below 50.0 kJ/mol [1,2,3,4,5], which is close to the energy required for the conformational transformation of molecules [6,7,8], indicating that there may be competition between proton transfer and conformational transformation under the external electric field. This has aroused great interest in exploring the reaction mechanisms induced by the external electric fields in chemistry, materials, biology, medicine, etc. [9,10,11,12].

The competition between the proton transfer and conformational transformation of drug molecules [13] stimulated by a bioelectric current, a kind of the electrical current generated by the cellular activity in nerves, muscles, and other tissues to maintain normal life activity [14,15,16,17,18,19], greatly affects the normal biochemical reaction process [20,21,22,23], such as the absorption, distribution, metabolism, and excretion of the drug. Thus, it determines the efficacy and toxicity of drugs [24,25,26,27]. Essentially, it is well known that competition and drug activity depend on the molecular structure of the drug and the internal environment of the organism. For example, if proton transfer dominates the expression of the drug activity, it requires an increase in drug polarity and a high concentration of ions in the internal environment; if the conformational transformation dominates the expression of drug activity, it requires the drug to be “soft” and the conformational transformation to be easy. Therefore, it is of great significance to clarify the competition between the proton transfer and conformational transformation of the drug molecules under the bioelectric current so as to reveal the essence of drug activity and develop new drugs.

**Scheme 1:**
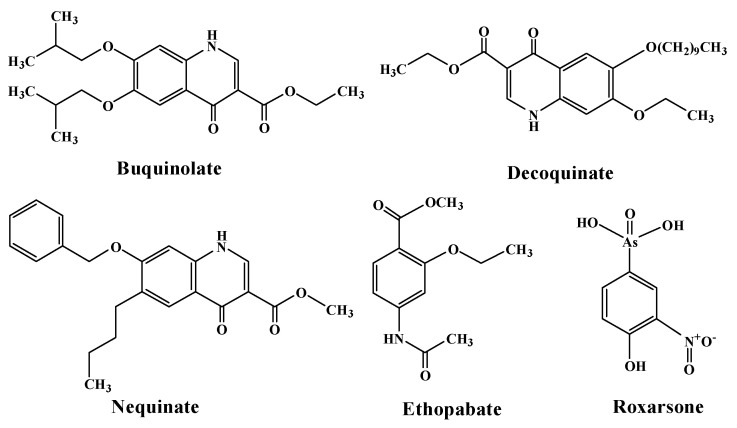
Structure of typical anticoccidial drugs.

**Figure 1:**
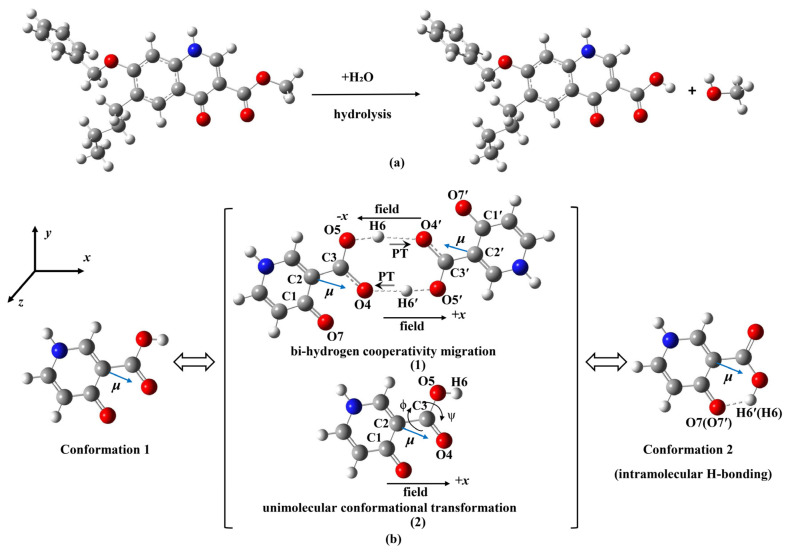
Hydrolysis of nequinate (**a**), proton transfer (PT) (**b**(1)) (intermolecular PT indicated by two arrows with “PT”) and conformation transformation (**b**(2)) of 4-pyridone-3-carboxylic acid under external electric field (coordinate axes parallel to which the external electric fields (*E_x_*, *E_y_*, *E_z_*) are applied), and the blue arrow labeled “*μ*” indicates the direction of the dipole moment.

Recently, anticoccidial drugs have attracted interest [28]. They can inhibit or kill parasitic coccidiosis by disrupting the cell membranes, interfering with the metabolic processes within cells and synthesis of coccidiosis DNA and RNA, etc. [29]. In particular, anticoccidial drugs with an aromatic ring containing adjacent –C=O carbonyl and –COOR ester groups (see Scheme 1) exhibit higher anticoccidiosis activity [30,31,32]. The corresponding mechanism of anticoccidiosis is as follows [33,34,35,36]: due to the electron transport driven by the intermolecular double-proton transfer between the –COOH groups from ester hydrolysis (see Figure 1a, nequinate as an example), the electron transport of the cytochrome system within the mitochondria of coccidiosis is disrupted, resulting in the inhibition of the growth and reproduction of sporophytes during the first asexual reproduction period of coccidiosis. Clearly, the products obtained from the intermolecular double-proton transfer and single-molecule conformational transformation by the rotation of the –COOH are the same, see Figure 1b. A question arises: is the mechanism of anticoccidiosis proton transfer or conformational transformation under a bioelectric current? Furthermore, is there competition between them?

Due to the technical difficulties in measuring and characterizing bioelectric effects, theoretical investigation has been favored to simulate the structures and properties of drug molecules under an external electric field [37]. There have been numerous quantum chemical methods used to reveal the essence of the proton transfer [38,39,40,41,42,43]. As for the conformation transformation, potential-energy–surface-scanning [44,45,46] has been the most commonly used method in the past few decades [47,48]. Recently, as a high-precision enhanced sampling rare event method, finite temperature string (FTS) has been favored in searching the path of the conformation transformation by the rotation of a chemical bond [49,50,51,52] due to the advantage of accurately describing the lowest free energy path [53]. Recently, Ren et al. [54,55] found that the FTS method with orientation order parameters (OPs) could make the free energy converge quickly.

In this work, 4-pyridone-3-carboxylic acid was selected as the modeling molecule for anticoccidial drugs, since, as mentioned above, the mechanism of anticoccidiosis is mainly related to the aromatic ring structure containing the adjacent –C=O and –COOH groups (see Figure 1). Double-proton transfer and conformational transformation were investigated by quantum chemistry calculations and FTS methods under an external electric field, respectively. The solvent effect of H_2_O on double-proton transfer was evaluated. This work is useful for providing a theoretical basis for exploring the essence of the anticoccidiosis of anticoccidial drugs under bioelectric currents, and further the selection and updates of anticoccidial drugs.

## 2. Theory

### 2.1. Finite Temperature String (FTS)

FTS is a computational method used to study rare events, such as phase transition and conformation transformation of complex systems. Via FTS, the reaction tubes, transition states, and transition paths on the free energy surface can be obtained by characterizing the changes in the collective coordinates, and thus the evolution mechanism of the system between different metastable states could be revealed [52,53].

For example, for a string $z\left(\alpha ,t\right)$ (*t* is time and $\alpha \in \left[0,1\right]$ as the string evolution parameter), it can converge to the minimum free-energy path (MFEP) [56]:

(1) $$\frac{𝜕Z_{i}\left(\alpha ,t\right)}{𝜕t}=-\sum_{j,k=1}^{N}P_{ij}\left(\alpha ,t\right)M_{jk}\left(z\left(\alpha ,t\right)\right)\frac{𝜕F\left(z\left(\alpha ,t\right)\right)}{{𝜕}_{Z_{k}}}$$

where $P_{ij}\left(\alpha ,t\right)$ represents the projection perpendicular to the string plane at $z\left(\alpha ,t\right)$, $\frac{𝜕F\left(z\left(\alpha ,t\right)\right)}{{𝜕}_{Z_{k}}}$ is the free-energy gradient (i.e., mean force), and $M_{jk}\left(z\left(\alpha ,t\right)\right)$ is the corresponding tensor. After the convergence of string, the MFEPs could be determined with the string method in collective variables [54,56].

### 2.2. Polarization and Stark Effect of Molecules Under External Electric Field

Under an external electric field *E*, molecules can be induced to polarize, generating induced dipole moments $\mu_{ind}$

(2) $${\bar{\mu }}_{ind}=\alpha \bar{E}$$

where *α* is the polarizability. The interaction induced by the dipole moment is [57]

(3) $$H_{a}=-\frac{1}{2}\left(\alpha_{∥}-\alpha_{⊥}\right)E^{2}\Phi^{2}-\frac{1}{2}\alpha_{⊥}E^{2}$$

where Φ is the cosine of the angle between the spatial coordinate and principal axis coordinate.

The interaction between the dipole moment and external electric field causes a change in the energy level of molecules, which is called the “Stark effect”. The first-order Stark effect *H_stark_* is

(4) $$H_{Stark}=-\bar{\mu }\cdot \bar{E}=-\mu E\cos\theta =-\mu EP_{1}\left(\cos\theta \right)$$

where *P*_1_(*cosθ*) is the first-order Legendre polynomial. The corresponding Stark energy is

(5) $$W_{Stark}^{\left(1\right)}=-\mu E\langle J,K,M\left|P_{1}\right|J,K,M\rangle =-\mu E\frac{MK}{J\left(J+1\right)}$$

The second-order Stark energy is

(6) $$W_{Stark}^{\left(2\right)}=\frac{1}{2}\mu E\Omega \left[\frac{\left(J^{2}-K^{2}\right)\left(J^{2}-M^{2}\right)}{J^{3}\left(2J-1\right)\left(2J+1\right)}-\frac{\left[{\left(J+1\right)}^{2}-K^{2}\right]\left[{\left(J+1\right)}^{2}-M^{2}\right]}{{\left(J+1\right)}^{3}\left(2J+1\right)\left(2J+3\right)}\right]$$

where *J*, *K*, and *M* are the angular momentum, projection of *J* in the direction of the external electric field, and molecular axis, respectively. *Ω* is a constant in a specific external electric field.

## 3. Results and Discussion

4-pyridone-3-carboxylic acid has two typical conformations, see Figure 1. In conformation 1, two carbonyl groups are on the same side. As for conformation 2, two carbonyl groups are on opposite sides, and an intramolecular H-bond forms between the hydroxyl and carbonyl group on the same side, with a bond distance of 2.26 Å at the M06-2X/aug-cc-pVTZs level. Conformation 2 is more stable than conformation 1, with an energy difference of 52.5 kJ/mol at the M06-2X/aug-cc-pVTZs level (see Figure S1).

### 3.1. Cooperativity Transfer of Intermolecular Double-Proton Under External Electric Field

For the dimer of 4-pyridone-3-carboxylic acid, there is a transition state corresponding to the intermolecular double-proton transfer, see Figure 1b. Under the absence of an external electric field, the barrier height (Δ*G*) of the intermolecular double-proton transfer is lower than that of the corresponding intramolecular proton transfer for the 4-pyridone-3-carboxylic acid monomer, exhibiting intermolecular double-proton cooperativity transfer characteristics. For example, at the M06-2X/aug-cc-pVTZs level, the barrier heights are 20.3 and 25.8 kJ/mol for the intermolecular and intramolecular proton transfer, respectively. Cooperativity double-proton transfer was also found in carboxylic derivatives (phthalic acids), and the barriers were calculated to be 0.7 and 0.9 kcal/mol (i.e., 2.9 and 3.8 kJ/mol) for proton transfer in the O···H–N intermolecular H-bond for the 3-nitrophthalic acid cocrystals with 2,4,6-collidine and N,N-dimethyl-4-pyridinamine at the B3LYP-D3/6-311+G(d,p) level, respectively [58]. The barrier height of the cooperativity intramolecular O···H–O proton transfer was 11.7 kcal/mol (i.e., 48.9 kJ/mol) for nitro-phthalic acid at the B3LYP-D3/6-311+G(d,p) level [59]. At the same level of theory, the barrier height of the intramolecular O···H–O proton transfer from the hydroxyl group of –COOH to the carbonyl group (i.e., the O7 atom, see Figure 1) in 4-pyridone-3-carboxylic acid was 33.8 kJ/mol, smaller than that of nitro-phthalic acid. It is noted that the negative charge of the O atom in carbonyl groups (i.e., the O7 atom) is greater than that of the O atom in the –COOH groups. Therefore, as a positively charged H atom, it is more likely to transfer to the O atom in the carbonyl group. In nitro-phthalic acid, O···H–O proton transfer occurs between two –COOH groups.

#### 3.1.1. Structure of Transition State

Table S1 provides the optimized geometrical parameters, AIMs (atoms in molecules), and surface electrostatic potential results for the transition state of the intermolecular double-proton transfer in the dimer of 4-pyridone-3-carboxylic acid along the different external electric fields (and those in the absence of field) at the M06-2X/6-311++G** and M06-2X/aug-cc-pVTZ levels. The bond lengths or AIM electron densities calculated at two theoretical levels are close, with a difference of less than 0.015 Ǻ or 0.02 a.u., respectively. This result indicates that it is feasible to use the M06-2X/6-311++G** method with higher computational efficiency to search for the transition state of the double-proton cooperativity transfer under external electric fields compared with the expensive M06-2X/aug-c-pVTZ method.

Under external electric fields, when the H6∙∙∙O4′ and H6′∙∙∙O4 distances increase, the distances of H6∙∙∙O5 and H6′∙∙∙O5′ decrease. In addition, due to the fact that the direction of the external electric field along the *x*- or *y*-axis is almost parallel to the plane of the carboxylic group, they have a significant influence on the changes in the H∙∙∙O distances in the transition state, while the direction of the external electric field along the *z*-axis is perpendicular to it, and thus it has little effect on the H∙∙∙O distance variation. Therefore, in this work, only the variation in the H6∙∙∙O4′ distance under the external electric fields along the *x*- and *y*-axis directions was considered.

Due to the fact that the H6∙∙∙O4′ direction is just along the *x*-axis, i.e., the approximate direction of the dipole moment [43] (see Figure 1), the influence of the external electric field along the *x*-axis on the change in the H6∙∙∙O4′ distance is more significant than that along the *y*-axis. For example, at the M06-2X/aug-cc-pVTZ level, the maximum decrease in the H6∙∙∙O4′ distance caused by the external electric field along the +*x*-direction is up to 0.051 Ǻ, while the maximum change along the +*y*-direction is less than 0.031 Ǻ.

At two levels of theory, the external electric field along the +*x* or +*y*-direction increases the H6∙∙∙O4′ distance, tending to form a double-proton cooperativity transfer product from conformation 2 to 1. The direction of the external electric field along the +*x* or +*y*-direction is opposite to the polarity direction of the O5–H6 chemical bond, and the electric field generated inside the molecule in the opposite direction to the external electric field is enhanced (i.e., the induced electric field generated by external electric field), resulting in an increase in the negative charge of O5 and the positive charge of H6. Thus, the O5–H6 bond is strengthened and it is difficult for H6 to transfer to the O4′ atom, leading to an increase in the H6∙∙∙O4′ distance. On the contrary, the external electric field along the −*x* or −*y*-direction decreases the H6∙∙∙O4′ distance, tending to form conformation 2. There are some good linear relationships between the change in the H6∙∙∙O4′ distance (Δ*R*) and external electric field strength (*E*) at both levels (see Figure S2), with the correlation coefficient *R*^2^ value exceeding 0.9800, indicating that the H6∙∙∙O4′ distance in the transition state is greatly influenced by the external electric field. In previous studies [38], there has also been a good linear relationship between the changes in the structures of the transition states and the strengths of the external electric fields. Similarly, the H6′∙∙∙O4 distance is also greatly influenced by the external electric field, and the external electric field along the +*x* or +*y*-direction decreases the H6′∙∙∙O4 distance as it has the same direction as the polarity direction of the O5′–H6′ chemical bond.

#### 3.1.2. AIM Analysis for Transition State

In order to reveal the essence of the H6∙∙∙O4′ distance variation in the transition state under the external electric field, an AIM analysis of H6∙∙∙O4′ is carried out under the external electric field (see Table S1). At two theoretical levels, as the strength of the external electric field changes, the change in the electron density *ρ* value of H6∙∙∙O4′ along the *x*-axis direction is obvious, while the influence of the external electric field along the *y*-axis direction on it is small, which is consistent with the result of structural changes. The external electric field along the +*x* or +*y*-direction decreases the electron density of H6∙∙∙O4′. On the contrary, in most cases, the external electric field along the −*x* or −*y*-direction increases the corresponding electron densities. According to Bader′s AIM theory [60], the higher the electron density at the critical point between atoms, the shorter the distance between them, and the stronger the interactions, leading to the potential formation of a new chemical bond (such as the H6–O4 bond) from an old intermolecular H-bond (such as H6∙∙∙O4′); On the contrary, the lower the electron density of AIM, the longer the distance, and the weaker the interaction strength, leading to the potential formation of a new intermolecular H-bond from an old chemical bond. Moreover, the greater the change in the electron density, the more significant the change in the strength of the interactions. In this way, according to the change in the AIM electron density, a double-proton cooperativity transfer product from conformation 2 to 1 can be formed under the external electric field along the +*x*-axis or +*y*-axis. On the contrary, a double-proton cooperativity transfer product from conformation 1 to 2 can be formed under the external electric field along the −*x*-axis or −*y*-axis, which is consistent with the structural changes. Similar to the change in the bond lengths, there were some good linear relationships between the changes in densities (Δ*ρ*) and electric field strengths (*E*). Figure S2 gives some linear relationships with correlation coefficient *R*^2^ values greater than 0.9800, showing that the AIM results of the H6∙∙∙O4′ distance in the transition state are greatly influenced by the external electric field.

#### 3.1.3. Electrostatic Potentials

The electrostatic potential (ESP) is closely related to the electrophilic (or nucleophilic) ability of the activated atoms in reactions and plays an important role in revealing the nature of reactions [61]. To further reveal the essence of the changes in the electronic structures of the transition states under the external electric field, the surface ESPs were analyzed. The calculated ESPs and corresponding statistical results are shown in Table S1. In each molecule of each electric field, there are several surface electrostatic potential minima (${\bar{V}}_{s}^{-}$) associated with the O atoms and surface ESP maxima (${\bar{V}}_{s}^{+}$) associated with the H atoms. For example, in the absence of an external electric field, the ${\bar{V}}_{s}^{-}$ of the carbonyl O4′ atom and the ${\bar{V}}_{s}^{+}$ of the H6 atom are −25.2 and 42.8 kcal/mol at the M06-2X/6-311++G** level, respectively.

Along the *x*-direction, as the electric field strength increases, the ${\bar{V}}_{s}^{+}$ value of H6 atom increases. Although the electric field along the *y*-direction has little effect on the *V*_S,max_ value of the H6 atom, most of the values also increase with the increase in the external electric field strength. This shows that, as the electric field strength increases along the *x*- and *y*-directions, the electrophilic ability of the H6 atom is strengthened in the proton transfer reaction. The trend of the changes in the ${\bar{V}}_{s}^{-}$ value is not obvious under an electric field.

In general, the electric field along the direction of the dipole moment (i.e., from the positive charge to the negative charge) enhances the dipole moment of molecule, resulting in an increase in the ${\bar{V}}_{s}^{+}$ value. The electric field opposite to the direction of the dipole moment weakens the dipole moment of the molecule, resulting in a decrease in the ${\bar{V}}_{s}^{+}$ value. From Figure 1, it can be seen that, for the 4-pyridone-3-carboxylic acid, the electric field along the +*x*-direction is the same as the dipole moment direction, and the ${\bar{V}}_{s}^{+}$ value of H6 atom increases. Similarly, for electric fields along the −*x*-direction, the polarization degree of H6∙∙∙O4′ decreases, resulting in an overall trend of a decrease in the ${\bar{V}}_{s}^{+}$ values of H6 atoms.

For the statistical measure of the surface ESPs, the effect of the external electric field is obvious. At two levels of theory, in most cases, the external electric field along the +*x*- or −*y*-direction decreases the value of $\sigma_{+}^{2}$. On the contrary, that along the −*x*- or +*y*-direction increases the value of $\sigma_{-}^{2}$. These results indicate that the statistical value of the surface ESP is significantly influenced by the external electric field. Furthermore, along the +*x*- and −*y*-directions, the electrophilic ability of the H6 atom is weakened in the proton transfer reaction. Along the −*x*- and +*y*-directions, the nucleophilic ability of the O4′ atom is strengthened.

Linear relationships were found between the surface electrostatic potentials ${\bar{V}}_{s}^{+}$, ${\bar{V}}_{s}^{-}$, statistical differences Δ$\sigma_{+}^{2}$, Δ$\sigma_{-}^{2}$, and external electric field strengths, with a correlation coefficient *R*^2^ value greater than 0.9500 (see Figure S2). This indicates that the surface ESP statistic result is greatly influenced by the external electric field.

#### 3.1.4. Frequency

The imaginary frequencies of the double-proton cooperativity transfer of the transition state under the external electric field are listed in Table 1. The effect of an external electric field along the *x*-axis on the imaginary frequency is much more significant than that along the *y*-axis. For example, the maximum reduction in imaginary frequency caused by the external electric field along the *x*-axis direction is 234.2 cm^−1^, while that along the *y*-axis direction results in a maximum imaginary frequency variation of less than 100.0 cm^−1^. This indicates that the effect of the external electric field along the *x*-axis direction on the double-proton transfer is more significant than that along the *y*-axis direction. Figure S3 shows several linear relationships between the changes in the frequencies and external electric field strengths, with correlation coefficient *R*^2^ values greater than 0.9000.

#### 3.1.5. Barrier Heights of Transition State and Rate Constant

The barrier heights for the double-proton cooperativity transfer under the external electric fields were calculated using the M06-2X/6-311++G**, M06-2X/aug-cc-pVTZ, and CCSD(T)/aug-cc-pVTZ methods (see Table 1). The calculated results from the three levels are close to each other, and the largest difference is not more than 10.0 kJ/mol.

At three levels of theory, as the field strength increases along the +*x* and −*y* directions, the energies of the transition state TS and reactant (i.e., conformation 1) decrease. The decrease in energy of the transition state is more pronounced, meaning that the external electric field has a greater influence on stabilizing the transition state than the reactants, leading to the decreased barrier heights. Consistent with the results of the molecular structure, electronic structure, and frequency analysis, the influence of the electric field along the +*x* direction on the barrier heights is much higher than that along the −*y* direction. At the CCSD(T)/aug-cc-pVTZ//M06-2X/aug-cc-pVTZ and M06-2X/aug-cc-pVTZ levels, compared with the barrier height without an electric field, the barrier heights along the +*x* (41.14 × 10^8^ Vm^−1^) direction decreased by 11.6 and 1.7 kJ/mol, respectively, with relative decreases of 67.1% and 10.6%, respectively. The electric field along the −*y* (41.14 × 10^8^ Vm^−1^) direction decreased the barrier heights by 3.0 and 2.0 kJ/mol, with relative values of 17.3% and 12.5%, respectively.

Along the −*x* and +*y* directions, as the field strength increased, the energy of the transition state and reactant increased, and the stability decreased. However, the energy of the transition state increased more significantly, meaning that the external electric field had a greater influence on stabilizing the reactants than the transition state, reducing the possibility of product formation and increasing the barrier heights. This is also consistent with the results of molecular structure, electronic structure, and frequency analysis. The effect of an electric field along the −*x* direction on the barrier was much higher than that along the +*y* direction. At the CCSD(T)/aug-cc-pVTZ//M06-2X/aug-cc-pVTZ and M06-2X/aug-cc-pVTZ levels, compared with the barrier height without an electric field, the electric field along the −*x* (41.14 × 10^8^ Vm^−1^) direction increased the barrier heights by 2.6 and 2.0 kJ/mol, respectively, while the relative values decreased by 15.0% and 12.5%. The effects of the electric field along the +*y* direction on the barrier were 2.1 and 1.8 kJ/mol, with a relative value of less than 12.1% and 11.3%.

There were some good linear relationships between the changes in barrier heights (Δ*G*) (compared with the barrier without an electric field) and the external electric field strengths (see Figure S3), with correlation coefficient *R*^2^ values greater than 0.9000. Compared with the correlation between the molecular structure or electronic structure changes and external electric field strengths, the correlation between the changes in the barrier heights and electric field strengths was weaker. This is because the change in barrier heights was closely related to both the reactants and transition states, and the change in energy is not only related to the molecular structure, but also closely related to the conformation. Numerous factors resulted in the weak linear correlations in the changes in barrier heights with field strength.

From Table 1, it can be seen that, consistent with the results of the molecular structure, electronic structure, frequency, and barrier heights, the influence of the external electric field along the +*x* direction on the rate constant was much higher than that along the −*y* direction. As mentioned above, along the +*x* and −*y* directions, as the field strength increased, the barrier decreased. Compared with the absence of an external electric field, the rate constant should increase. However, along the +*x* or −*y* direction, as the field strength increased, some of the rate constants decreased. This is because the external electric field along the +*x* or −*y* direction had little effect on the change in the free energy of the reaction. Moreover, in the external electric field, the tunneling effect of the reaction, *κ*_298.15K_, underwent significant changes. In the absence of an external electric field, the value of the tunneling effect *κ*_298.15K_ is 2.18, while in the −*y* direction of the external electric field, most of the values exceed 2.33, leading to a significant change in the rate constant. This explains why the change trends of the rate constants were not consistent with those of the barrier heights under the external electric fields. Therefore, the tunneling effect cannot be ignored for the double-proton cooperativity transfer of the 4-pyridone-3-carboxylic acid dimer under the external electric fields. Similar results have also been observed for the rate constant variations along the −*x* and +*y* directions.

Under the external electric field along the +*x* direction, the barrier height of the double-proton cooperativity transfer was low and the rate constant was large, even up to about 1.0 × 10^16^ (see 41.14 × 10^8^ Vm^−1^ (+*x*)), showing that it was prone to occur. Therefore, it can be predicted that, under the bioelectric current, the double-proton cooperativity transfer has a significant influence on the drug activity of 4-pyridone-3-carboxylic acid. It should be noted that the present work employs some excessively strong electric fields that are not present under biological conditions, but that could be employed potentially at the wet-lab level.

#### 3.1.6. Solvation Effect

The solvation effects influence remarkably the structural, electronic, vibrational, and chemical properties of the solute molecules [62]. In order to clarify the kinetics in solution, the solvent effects of H_2_O on the intermolecular cooperativity double-proton transfer were evaluated by the integral equation formalism polarized continuum model (IEFPCM) [63,64] based on the self-consistent reaction field (SCRF) [65] at the M06-2X/aug-cc-pVTZ (geometry optimization) and CCSD(T)/aug-cc-pVTZ//M06-2X/aug-cc-pVTZ (dynamic calculation) levels of theory along the *x*-axis direction with the electric field strengths of ±41.14 × 10^8^ Vm^−1^.

Table S1 provides the optimized geometrical parameters, AIM, and surface electrostatic potential results in aqueous solution for the transition state of the proton transfer in the dimer of 4-pyridone-3-carboxylic acid at two levels of theory.

Under the absence of an external electric field, the values of *R*_H6∙∙∙O4′_ in aqueous solution were larger than those in the gas-phase structure, while the values of *ρ*_H6∙∙∙O4′_ in aqueous solution were lower than those in the gas-phase structure. These results show that, from the gas phase to the solution phase, the proton transfer becomes difficult from conformation 1 to conformation 2. Similar to the gas phase, the external electric field along the +*x*-direction increased the H6∙∙∙O4′ distance or decreased the electron density of H6∙∙∙O4′ at two levels of theory, tending to form a double-proton cooperativity transfer product from conformation 2 to 1. On the contrary, the external electric field along the −*x*-direction decreased the H6∙∙∙O4′ distance or decreased the electron density of H6∙∙∙O4′, tending to form a double-proton cooperativity transfer product from the conformation 1 to 2.

For the surface electrostatic potentials, there was no obvious difference between the gas and solvent phase. The absolute values of the most negative ${\bar{V}}_{s}^{-}$ or most positive ${\bar{V}}_{s}^{+}$ increased slightly from the gas phase to the solution phase under the absence of an external electric field at two levels of theory. The values of $\sigma_{+}^{2}$ and $\sigma_{-}^{2}$ decreased slightly from the gas phase to the solution phase. Under the external electric field, the pattern was not clear for the change of the surface electrostatic potentials; some were increased and some are decreased.

From Table 1, under the absence of an external electric field, the values of ν decreased, and they also decreased from the gas to solvent phase along the +*x*- or −*x*-direction. From the gas to solvent phase, the barrier heights (Δ*G*_298.15K_) increased at the M06-2X/6-311++G**, M06-2X/aug-cc-pVTZ and CCSD(T)/aug-cc-pVTZ//M06-2X/aug-cc-pVTZ levels, leading a decreased reaction rate constant (*k*_298.15K_) of the double-proton transfer in the absence of field, as is in agreement with the result from the structures. Biswas et al. suggested that the incomplete transfer of the proton from water to the aniline anion showed the increased energy barrier caused by the solvation effect from surrounding water molecules [66]. Under the external electric field along the +*x*- or −*x*-direction, the barrier heights also increased, and the reaction rate constants also decreased.

### 3.2. FTS of Conformational Transformation Under External Electric Field

#### 3.2.1. Initial String and Convergence

Kulshrestha et al. [67] validated the FTS method in studying the conformational change by alanine dipeptide as an excellent test in a vacuum. The computed free-energy landscape shows excellent agreement with the previously published results obtained from other methods, including the quantum mechanics and molecular dynamics methods. Therefore, in this work, the FTS method was used to investigate the conformational transformation of 4-pyridone-3-carboxylic acid under the external electric field.

In order to determine the structures of two stable conformations in the initial string, the dihedral angles *ϕ* and *ψ* as the OPs, which are closely related to the conformational transformation of 4-pyridone-3-carboxylic acid (see Figure 1b), are evaluated by root-mean-square deviations (RMSD). The result shows that the differences in the *ϕ* and *ψ* values between the two stable conformations were 162.7° and 158.3°, consistent with the results obtained at the M06-2X/aug-cc-pVTZ level (168.5° and 153.0°, respectively). Therefore, in order to perform umbrella sampling, 18 windows were selected and the differences in the *ϕ* and *ψ* values were divided into 18 equal values; thus, the initial string was constructed.

The quality of the initial string is crucial for the convergence of the string. In order to obtain a good initial string, the string from conformation 1 to 2 was divided into 20 equally partitioned independent spaces, including 2 stable states and 18 intermediate conformations. Then, a MD simulation was performed on the initial string in no field, and along the +*x*-direction with the electric field strengths of 10.28 × 10^8^ Vm^−1^, 20.56 × 10^8^ Vm^−1^ and 30.84 × 10^8^ Vm^−1^, and along the *x*-, *y*-, and *z*-directions under the maximum electric field strengths (i.e., +41.14 × 10^8^ Vm^−1^ (*x*), +41.14 × 10^8^ Vm^−1^ (*y*), and +41.14 × 10^8^ Vm^−1^ (*z*)), obtaining 10 ns trajectories for each direction.

**Figure 2:**
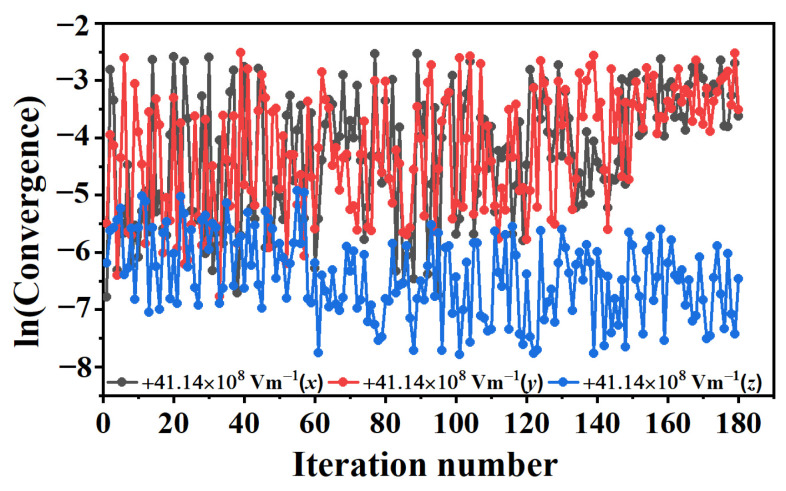
Convergence of the collective variables during string evolution under the different external electric fields.

In order to evaluate the effectiveness of the algorithm, the convergence was investigated. Convergence is an important indicator for measuring rare event methods. For the conformational transformations of the complex systems, in general, the rare event method required no more than 100 iterations to converge [39,68,69]. With the umbrella sampling and parameter averaging algorithm, the trajectory obtained in the *x*-direction converged after about 40 iterations with the field strengths of +41.14 × 10^8^ Vm^−1^ (see Figure 2), while the trajectories obtained in the *y*- and *z*-directions converged after about 60 and 70 iterations, respectively.

#### 3.2.2. Potential of Mean Force (PMF)

**Figure 3:**
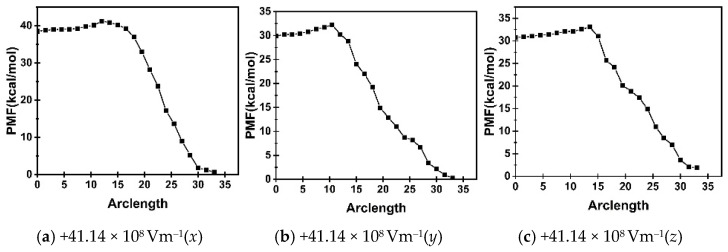
PMF of the conformational transformation of 4-pyridone-3-carboxylic acid under the different external electric fields.

**Figure 4:**
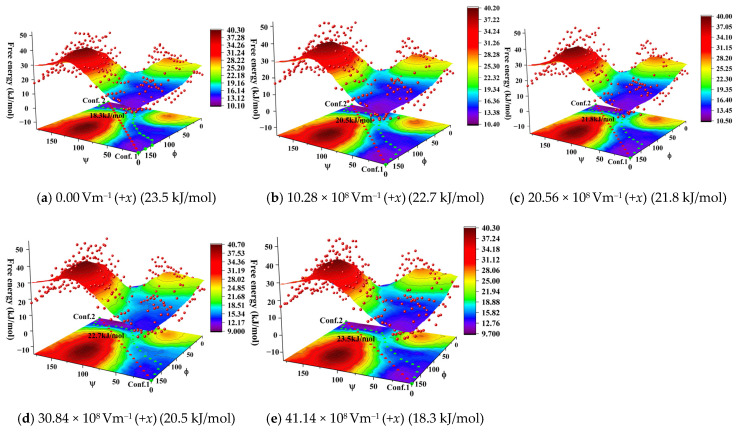
3D surface plot of the free energy landscape with transition states from conformation 1 to 2 under the different external electric fields.

The PMF is the line integral of the restraint force along the path, and it shows the MFEP as the maximum-likelihood path in the space of collective variables along FTS [52,53,56]. On the basis of the convergence judgment and string smoothing processing, the 2D graph and potential energy surface of the PMF along the path of the conformational transformation of 4-pyridone-3-carboxylic acid calculated using the FTS method are shown in Figure 3. From Figure 3a, it can be seen that there is a free-energy barrier from conformation 2 to conformation 1, with the (*ϕ*, *ψ*) values of OPs ranging from approximately (40.0°, 100.0°) to (60.0°, 80.0°) (see Figure 4).

From Figure 3a–c, it can be seen that the PMF curves of the conformational transformation of 4-pyridone-3-carboxylic acid with the electric field strength of 41.14 × 10^8^ Vm^−1^ were almost identical along the three different directions of the +*x*-, +*y*-, and +*z*-axes, and the two maximum values were also almost equal. This indicates that, during the conformational transformation, all the PMFs along the FTS pathway showed the same change trends with respect to (*ϕ*, *ψ*), and the direction of the external electric field was insensitive to the spatial OPs (*ϕ*, *ψ*) and PMFs. This is because the dipole moment of the 4-pyridone-3-carboxylic acid molecules did not change significantly during the conformational transformation, and did not involve significant redistribution of charges within the molecule. During the conformational transformation process, the changes in two angles were not synchronized under an external electric field. For example, when the change in *ψ* exceeded 150.0°, that of *ϕ* was less than 50.0°.

#### 3.2.3. Free Energy Landscape

In order to obtain the free-energy changes during the conformational transformation process, smoothing string was carried out for the initially converged strings. For the FTS method, each representative point along the path could be considered as an intermediate state in the conformational transformation process, and a smooth string meant that these intermediate states could continuously change from one end to the other, while the purpose of smoothing the string was to eliminate irrelevant information in the string evolution process. As mentioned above, the direction of the external electric field was insensitive to the spatial OPs (*ϕ*, *ψ*) and PMFs, and thus only the free energy landscape along the +*x*-axis direction was considered. The free-energy landscape in different external electric fields is shown in Figure 4.

According to Figure 4, the transformation between conformations 1 and 2 is a multi-step process involving the rotations around the C2–C3 and C3–O5 single bonds, as well as the formation of the intramolecular hydrogen bond. From conformation 1 to 2, the values of *ϕ* change from approximately 0° to 170°, accompanied by rotation around the C2–C3 bond, and those of *ψ* change from approximately 0° to 180°, corresponding to the rotation around the C3–O5 bond. The potential barriers (i.e., barrier of free energy) of the rotations around the C2–C3 and C3–O5 single bonds were up to about 20.0 kJ/mol. When the value of *ϕ* changed from approximately 150° to 100°, the change in the value of *ψ* did not exceed 10, indicating that the changes in *ϕ* and *ψ* were not synchronized during the conformational transformation. Similarly, when the value of *ψ* changed from approximately 110° to 160°, the change in the *ϕ* value did not exceed 5°, corresponding to the formation of the intramolecular hydrogen bonds. Moreover, during the formation of the intramolecular hydrogen bonds, the “3D surface plot” corresponded to blue, and the potential barrier for the formation of the intramolecular hydrogen did does not exceed 5.0 kJ/mol, which was smaller than the potential barrier for the entire conformational transformation process, approximately 25.0% of the potential barrier for the entire conformational transformation. Therefore, in this work, the formation process of the intramolecular hydrogen bonds was not considered.

The overall pathway depends on the external electric fields. As the electric field strength increased along the +*x*-axis direction, the potential barrier of the conformational transformation increased, but the increase was relatively small. The electric field strength increased from 0.0 to 41.14 × 10^8^ Vm^−1^, while the potential barrier only increased by less than 5.5 kJ/mol. Therefore, similar to the direction of the external electric field, the field strength was insensitive to the potential barrier of the conformational transformation.

In summary, from the FTS of the conformational transformation for 4-pyridone-3-carboxylic acid, it was found that the umbrella sampling and parameter averaging could quickly converge the OPs (*ϕ*, *ψ*), thereby obtaining the PMF for conformational transformation. This validates the effectiveness of the string method in the study of the conformational transformation of small molecules.

### 3.3. Comparison of Double-Proton Transfer and Conformation Transformation

Comparing Table 1 with Figure 4, under the external electric field, the barrier height of the double-proton cooperativity transfer of the 4-pyridone-3-carboxylic acid dimer is close to that for the conformational transformation. The barrier heights for the double-proton cooperativity transfer were between 5.0 and 20.0 kJ/mol, while the value of the conformational transformation was about 18.3 kJ/mol.

It should be noted that, above, the barrier of the conformational transformation was obtained from an empirical potential simulation based on the FTS method, which could not be compared with that of the proton transfer obtained from the quantum chemistry calculations. In order to evaluate how far the barrier heights from two processes change, two points on the MFEP, i.e., conformation 2 and the transition state with (*ϕ*, *ψ*) values of (7.5°, 168.3°) and (48.0°, 92.7°), were selected, and a constrained optimization was carried out at the M06-2X/6-311++G**, M06-2X/aug-cc-pVTZ, and CCSD(T)/aug-cc-pVTZ (only with the electric field strength of 0.0 Vm^−1^ and ±41.14 × 10^8^ Vm^−1^) levels. Then, the barrier heights were calculated with the M06-2X/6-311++G**, M06-2X/aug-cc-pVTZ, and CCSD(T)/aug-cc-pVTZ//M06-2X/aug-cc-pVTZ and CCSD(T)/aug-cc-pVTZ methods. Under the absence of an external electric field, the barrier heights were 21.4 and 16.3 kJ/mol at the CCSD(T)/aug-cc-pVTZ//M06-2X/aug-cc-pVTZ and CCSD(T)/aug-cc-pVTZ levels, and under the electric field strength of +41.14 × 10^8^ Vm^−1^ (*x*), they were 8.6 and 7.7 kJ/mol, respectively. The barrier heights with the conformational transformation were very close to those from the quantum chemistry calculations for the double-proton transfer at the same levels (i.e., 17.3, 15.2, 5.7, and 6.8 kJ/mol, see Table 1), with a maximum difference of less than 5.0 kJ/mol. This indicates that there may be competition between the proton transfer and conformational transformation under the external electric field. Therefore, in general, two mechanisms were both important for the drug, and they were both helpful in the selection of anticoccidial drugs. In order to increase the drug activity, polarity groups (such as –OH, –COOH, –C=O, etc.) should be added to make the proton cooperativity transfer easy, and the branched chain of the molecule should be increased to make the conformational transformation easy. However, it should be noted that ions are always involved in the metabolic processes of substances and energy in living organisms, which may increase the possibility of the double-proton cooperativity transfer.

The direction of the external electric field greatly affects the double-proton cooperativity transfer, but it has little effect on the conformational transformation. For the double-proton cooperativity transfer, the influence of the external electric fields along the *x*- and *y*-axis directions on the geometric structure, AIM results, surface electrostatic potentials, or barrier height of the transition state is significant. The influence of external electric fields along the *x*-axis direction parallel to the double-proton transfer is the most significant, mainly due to the significant dipole moment changes. From Equations (3) and (4) in “2. Theory”, it can be seen that the external electric field along the *x*-axis direction has a smaller value of *θ* and polarizability *α*_⟂_, and an larger value of *α*_∥_ or cos(*θ*) (i.e., Φ), resulting in an increase in the values of the interaction *H*_a_ and first-order Stark effect *H_stark_* induced by dipole moment. Furthermore, from Equations (5) and (6), it can be seen that, as the external electric field along the *x*-axis increases, the values of M and K (i.e., the projection of *J* in the direction of the external electric field and molecular axis) also increase, resulting in an increase in the value of the first-order and second-order Stark energies. These increase the changes in the geometric structure, AIM results, surface electrostatic potential and its statistics, or barrier height of the transition state under the external electric fields along the *x*-axis direction. Thus, some good linear relationships formed between the changes of the bond lengths, AIM results, surface electrostatic potentials, barriers of the transition state, and the external electric field strengths. It should be noted that, in practice, although the changes could occur in the presence of an electric field, its strong magnitude hinders its application in biological systems, such as the decomposition of drug molecules, increase in drug toxicity, etc.

For the conformational transformation process of 4-pyridone-3-carboxylic acid, due to the small change in dipole moment, there is no significant redistribution of charges in the molecule, and thus the direction of the external electric field had little effect on the (*ϕ*, *ψ*) and PMF values. Due to the fact that the direction of the conformational transformation (i.e., the rotations around C2–C3 and C3–O5 single bonds) is approximately perpendicular to the direction of the dipole moment, the external electric field has little effect on the interaction *H*_a_, first-order Stark effect *H_stark_*, first-order or second-order Stark energies, resulting in the insensitivity of the external electric field to the conformational transformations.

## 4. Calculation and Simulation Details

### 4.1. Chemical Kinetics Calculation Details

All the calculations were performed using Gaussian 09 packages [70]. All the reactants, transition states (TSs), and products were fully optimized at the M06-2X/6-311++G** and M06-2X/aug-cc-pVTZ levels in the external electric fields and in no field, and the global and local stable points on the potential energy surface were determined based on the frequency vibration modes. The barrier heights and reaction rate constants for each reaction were calculated at the M06-2X/6-311++G**, M06-2X/aug-cc-pVTZ, CCSD(T)/aug-cc-pVTZ//M06-2X/aug-cc-pVTZ, and CCSD(T)/aug-cc-pVTZ levels. The AIMs (atoms in molecules) results [71] and surface electrostatic potential [72] in the different external electric fields were obtained at the M06-2X/aug-cc-pVTZ levels.

In the selected coordinate system, the O5 atom of the –OH group in 4-pyridone-3-carboxylic acid is taken as the coordinate origin (see Figure 1). The H6 atom of the –OH group is located in the +*x*-axis direction, the *y*-axis is on the carboxylic acid plane, and “+*y*-axis” means the direction from the C3 to O5 atom, and it is perpendicular to the O5–H6 bond. The *z*-axis is perpendicular to the “*xy*” plane and the “+*z*-axis” means the direction from “outside” to “inside”. In the orthogonal directions of *x*, *y*, and *z*, the electric field strengths were chosen as ±5.14 × 10^8^ Vm^−1^, ±10.28 × 10^8^ Vm^−1^, ±15.42 × 10^8^ Vm^−1^, ±20.56 × 10^8^ Vm^−1^, ±25.70 × 10^8^ Vm^−1^, ±30.84 × 10^8^ Vm^−1^, ±35.97 × 10^8^ Vm^−1^, and ±41.14 × 10^8^ Vm^−1^, respectively.

The integral equation formalism polarized continuum model (IEFPCM) based on the self-consistent reaction field (SCRF) [63,64,65] with H_2_O (*ε* = 78.35) was employed for the calculations of geometry optimizations and intermolecular interaction energies at the M06-2X/aug-cc-pVTZ (geometry optimization) and CCSD(T)/aug-cc-pVTZ (dynamic calculation) levels of theory along the *x*-axis direction with electric field strengths of ±41.14 × 10^8^ Vm^−1^.

### 4.2. Simulation Details of FTS

Classical MD simulations were carried out for the conformational changes using FTS, and the FTS method was used to compute the transition pathway with the free-energy landscape determined through umbrella sampling simulations.

The initial structure of 4-pyridone-3-carboxylic acid under the external electric field was from the fully optimized results at the M06-2X/aug-cc-pVTZ level. The bond angle *ϕ* and dihedral angle *ψ* of the conformational transformation (*ϕ, ψ*) was selected as the collective coordinate (i.e., reaction coordinate), see Figure 1. For the initial string, 18 transition conformations with 18 windows (nodes) were constructed and optimized under the external electric field.

For the dynamics, firstly, a 3 × 3 × 3 nm^−3^ model box containing 81 molecules (i.e., 1215 atoms) of 4-pyridone-3-carboxylic acid was constructed. To prevent the influence of periodicity on the model boundary, the boundary distance between 4-pyridone-3-carboxylic acid molecules and the simulation box was set to 1.2 Å. Then, we used a time step of 0.1 fs and a Nosé–Hoover thermostat to maintain the temperature at 298 K. For bias potential umbrella sampling, the spring coefficients of the key angle *ϕ* and dihedral angle *ψ* variables as the OPs were set to be 100.0 kJ∙mol^−1^∙rad^−1^, and their evolution was estimated using the mean-value method. These potentials were used to perform restrained dynamics around each image of the string and compute the mean force. For each image on the string, both the mean force and tensor were computed as the ensemble average over 250 ps of molecular dynamics simulation. For each step of the FTS simulation, in order to maintain the stability of the simulation, the initial configuration of each step was chosen to be the same as the structure in which the rotation angle was close to that of the previous step. Moreover, the constraints on parameters in each step were obtained from the previous step. For the subsequent period, the initial velocity of each step was randomly assigned from the Maxwell–Boltzmann distribution of the temperature 273.15 K. The FTS simulation was calculated using the CHARMM22 force field [73] from the NAMD 2.11 software packages [74] and PLUMED program [75].

## 5. Conclusions

In this work, the kinetics of the intermolecular double-proton cooperativity transfer and FTS of the conformational transformation for 4-pyridone-3-carboxylic acid were investigated under the external electric field. The conclusion is as follows:

(1) Due to the first-order Stark effect induced by the dipole moment, the external electric fields parallel to the direction of the molecular dipole moment had a significant influence on the structures, AIMs, and surface ESPs of the transition states of the double-proton cooperativity transfer, accompanied by some significant linear relationships between them and the strengths of the external electric fields. The corresponding products were controlled by the direction of the external electric field. From the gas to solvent phase, the barrier heights increased.
(2) Umbrella sampling FTS and parameter averaging can quickly converge the OPs (*ϕ*, *ψ*), thereby obtaining the PMF and free-energy landscape of the conformational transformation.
(3) Under the external electric field, the barrier height of the intermolecular double-proton cooperativity transfer was close to that of the conformational transformation, indicating the competition between them. The external electric field affected greatly the double-proton transfer, while it had little effect on the free energy landscape of the conformational transformation.
(4) Two mechanisms are important for the drug, and they are both helpful in the selection of anticoccidial drugs. In order to increase the activity of anticoccidial drugs, polarity groups should be added to make the proton cooperativity transfer easy, and the branched chain of the molecule should be increased to make the conformational transformation to be easy.

This study is helpful in the selection and updating of anticoccidial drugs.

## Figures and Tables

**Table 1:** Transition states’ imaginary frequencies (Im(*v*), cm^−1^), barrier heights (∆*G*_298.15K_, kJ/mol), Wigner tunneling corrections (*κ*_298.15K_), and corrected reaction rate constants (*k*_298.15K_) of the double-proton transfer in the absence and presence of fields of varying strengths and directions (*x*- and *y*-directions) at the M06-2X/6-311++G**, M06-2X/aug-cc-pVTZ, and CCSD(T)/aug-cc-pVTZ levels.

Field (×10^8^ Vm^−1^)	Im(*v*) ^a^	∆*G*_298.15K_^a^	∆*G*_298.15K_^b^	∆*G*_298.15K_^c^	*κ*_298.15K_	*k*_298.15K_ ^c^
No field	1517.8 1426.3 ^d^	16.3 17.4 ^d^	16.0 18.2 ^d^	17.3 (15.2 ^e^) 17.4 ^d^	2.18	1.83 × 10^9^ 1.09 × 10^9 d^
41.14 (+*x*)	1283.6 1255.7 ^d^	13.6 14.7 ^d^	14.3 14.9 ^d^	5.7 (6.8 ^e^) 6.0 ^d^	1.71	6.32 × 10^16^ 4.89 × 10^16 d^
35.97 (+*x*)	1316.3	14.0	14.7	6.5	1.56	8.19 × 10^15^
30.84 (+*x*)	1388.9	14.5	15.0	6.8	1.27	2.06 × 10^15^
25.70 (+*x*)	1418.8	14.8	15.2	7.7	1.96	1.35 × 10^15^
20.56 (+*x*)	1452.2	15.3	15.3	8.5	1.87	7.29 × 10^14^
15.42 (+*x*)	1480.5	15.9	15.6	9.1	2.03	2.87 × 10^13^
10.28 (+*x*)	1496.3	15.9	15.7	11.3	2.11	3.91 × 10^11^
5.14 (+*x*)	1513.7	16.0	15.8	15.1	2.16	2.68 × 10^10^
5.14 (−*x*)	1528.2	16.4	16.2	17.4	2.21	1.66 × 10^9^
10.28 (−*x*)	1566.9	16.6	16.3	17.6	2.18	1.01 × 10^9^
15.42 (−*x*)	1593.1	17.0	16.7	17.9	2.16	9.89 × 10^8^
20.56 (−*x*)	1601.5	17.4	16.8	18.2	2.19	8.11 × 10^7^
25.70 (−*x*)	1623.7	17.8	17.2	18.4	2.32	6.3 × 10^7^
30.84 (−*x*)	1638.2	18.0	17.4	18.9	2.18	1.95 × 10^7^
35.97 (−*x*)	1671.6	18.6	17.5	19.6	2.15	7.78 × 10^6^
41.14 (−*x*)	1689.8 1652.7 ^d^	18.9 18.9 ^d^	18.0 18.8 ^d^	19.9 20.1 ^d^ (17.3 ^e^)	2.33	5.11 × 10^5^ 1.99 × 10^5 d^
41.14 (+*y*)	1566.7	14.1	14.2	19.4	2.57	8.52 × 10^6^
35.97 (+*y*)	1540.7	14.4	14.4	19.3	2.40	9.91 × 10^6^
30.84 (+*y*)	1532.7	14.7	14.6	18.4	2.51	6.96 × 10^7^
25.70 (+*y*)	1535.7	15.0	14.8	18.2	2.63	1.06 × 10^8^
20.56 (+*y*)	1563.5	15.5	15.3	18.0	2.28	8.85 × 10^8^
15.42 (+*y*)	1542.6	15.8	15.3	17.8	2.17	9.98 × 10^8^
10.28 (+*y*)	1523.8	16.0	15.8	17.5	2.15	2.66 × 10^9^
5.14 (+*y*)	1508.0	16.2	15.9	17.3	2.19	8.18 × 10^9^
5.14 (−*y*)	1506.6	16.3	16.3	17.1	2.26	9.25 × 10^9^
10.28 (−*y*)	1495.0	16.5	16.4	17.0	2.12	9.99 × 10^9^
15.42 (−*y*)	1492.5	16.9	16.6	16.9	2.18	1.01 × 10^10^
20.56 (−*y*)	1482.3	17.2	16.8	16.5	2.32	1.93 × 10^10^
25.70 (−*y*)	1473.6	17.3	17.0	16.2	2.05	2.01 × 10^10^
30.84 (−*y*)	1435.2	18.1	17.3	15.2	2.17	2.21 × 10^10^
35.97 (−*y*)	1426.8	18.3	17.8	15.2	2.33	2.97 × 10^10^
41.14 (−*y*)	1418.9	18.8	18.0	14.3	2.25	8.50 × 10^10^

^a^ At M06-2X/6-311++G** level; ^b^ At M06-2X/aug-cc-pVTZ level; ^c^ At CCSD(T)/aug-cc-pVTZ//M06-2X/aug-cc-pVTZ level; ^d^ In aqueous solution; ^e^ At CCSD(T)/aug-cc-pVTZ level.

## Data Availability

The data related to this research can be accessed upon reasonable request via email.

## Supplementary Materials

The following supporting information can be downloaded at: https://www.mdpi.com/article/10.3390/molecules30153115/s1. Table S1. The optimized transition state geometrical parameters and AIM results of the double-proton transfer reaction for 4-pyridone-3-carboxylic acid dimer, surface electrostatic potentials, and their statistical quantities in the absence and presence of fields of varying strengths and directions at the M06-2X/6-311++G** (in blank) and M06-2X/aug-cc-pVTZ (in bold) levels; Figure S1. Scheme of energy barriers on proton transfer and transformation for 4-pyridone-3-carboxylic; Figure S2. Relationships between the structures, AIMs, electrostatic potential results of TS, and external electric field strengths; Figure S3. Relationships between the frequencies, barrier heights, and external electric field strengths are in the Supporting Information.
