# Supplementary material for: Kinetics of Proton Transfer and String of Conformational Transformation for 4-Pyridone-3-carboxylic Acid Under External Electric Field
Source: Molecules. 2025 Jul 25;30(15):3115. doi: 10.3390/molecules30153115 (PMC12348352; doi:10.3390/molecules30153115)
Supplement: Supplementary file 1 [file molecules-30-03115-s001.zip › molecules-3710898-supplementary.pdf]

Supporting Information

Kinetics of Proton Transfer and String of Conformational Transformation for 4-pyridone-3-carboxylic Acid Under External Electric Field

Ya-Wen Li, Rui-Zhi Feng, Xiao-Jiang Li, Ai-Chuan Liu, En-Lin Wang

Table S1 The optimized transition state geometrical parameters and AIM results of the double proton transfer reaction for 4-pyridone-3-carboxylic acid dimer, surface electrostatic potentials (V_s^+, V_s^-, kcal/mol) and they statistical

quantities (σ_+^2 , σ_-^2 , kcal/mol) in the absence and presence of fields of

varying strengths and directions (*x*- and *y*-directions) at the M06-2X/6-311++G** (in blank) and M06-2X/aug-cc-pVTZ (in bold) levels.

Figure S1 Scheme of energy barriers on proton transfer and transformation for 4-pyridone-3-carboxylic

Figure S2 Relationships between the structures, AIM, electrostatic potential results of TS and external electric field strengths at the M06-2X/6-311++G** and M06-2X/aug-cc-pVTZ levels with different electric field directions (+*x*-, –*x*-, +*y*-,and –*y*-directions).

Figure S3 Selected relationships between the frequencies, barrier heights (ΔG) and external electric field strengths at the M06-2X/6-311++G** (i.e., M06-2X/6), M06-2X/aug-cc-pVTZ (i.e., M06-2X/aug) and CCSD(T)/aug-cc-pVTZ (i.e., CCSD(T)/aug) levels with different electric field directions (+*x*-, –*x*-, +*y*-,and –*y*-directions).

Table S1 The optimized transition state geometrical parameters and AIM results of the double proton transfer reaction for 4-pyridone-3-carboxylic acid dimer, surface electrostatic potentials ($\overline{V}_s^+$, $\overline{V}_s^-$, kcal/mol) and they statistical quantities (σ_+^2 , σ_-^2 , kcal/mol) in the absence and presence of fields of varying strengths and directions (*x*- and *y*-directions) at the M06-2X/6-311++G** (in blank) and M06-2X/aug-cc-pVTZ (in bold) levels.

Field(×10^8 Vm^{-1})	$R_{H6\cdots O4'}$ (Å)	$\rho_{H6\cdots O4'}$(a.u.)	$\overline{V}_s^+$	$\overline{V}_s^-$	σ_+^2	σ_-^2
No field	1.3420	0.1611	42.8	–25.2	293.7	69.11
	**1.3412**	**0.1695**	**38.2**	**–21.3**	**269.3**	**58.67**
	1.3457 [a]	*0.1583*	*43.9*	*–27.1*	*290.3*	*67.24*
	1.3468 [a]	***0.1613***	***40.0***	***–23.5***	***263.5***	***56.93***
41.12 (+*x*)	1.3925	0.1163	63.2	–37.2	351.2	55.13
	**1.4058**	**0.1332**	**66.6**	**–33.5**	**335.7**	**58.17**
	1.3513 [a]	*0.1126*	*62.8*	*–35.3*	*346.8*	*53.56*
	1.3527 [a]	***0.1268***	***65.7***	***–31.6***	***338.2***	***55.28***
35.97 (+*x*)	1.3878	0.1215	59.5	–35.6	346.1	57.10
	**1.3967**	**0.1396**	**59.3**	**–32.8**	**331.3**	**61.37**
30.84 (+*x*)	1.3819	0.1287	56.8	–34.3	341.7	58.12
	**1.3822**	**0.1413**	**54.2**	**–30.2**	**326.6**	**63.28**
25.70 (+*x*)	1.3723	0.1318	53.6	–33.5	336.5	61.33
	**1.3793**	**0.1479**	**50.3**	**–29.3**	**319.5**	**65.19**
20.56 (+*x*)	1.3685	0.1395	51.0	–31.2	327.2	62.98
	**1.3741**	**0.1521**	**47.2**	**–28.1**	**306.2**	**62.26**
15.42 (+*x*)	1.3622	0.1467	48.8	–29.6	318.3	65.13
	**1.3609**	**0.1597**	**43.5**	**–27.2**	**298.6**	**63.18**
10.28 (+*x*)	1.3516	0.1536	45.7	–28.3	305.1	68.22
	**1.3532**	**0.1622**	**41.8**	**–25.8**	**286.5**	**60.23**
5.14 (+*x*)	1.3423	0.1582	43.2	–27.7	294.8	69.13
	**1.3415**	**0.1663**	**39.5**	**–22.2**	**270.1**	**58.11**
5.14 (–*x*)	1.3388	0.1642	41.6	–24.5	291.5	69.02
	**1.3352**	**0.1713**	**38.3**	**–20.2**	**269.1**	**58.13**
10.28 (–*x*)	1.3335	0.1668	42.2	–23.5	288.6	68.13
	**1.3263**	**0.1808**	**41.0**	**–19.9**	**266.8**	**57.37**
15.42 (–*x*)	1.3267	0.1757	45.6	–23.3	285.3	67.22
	**1.3158**	**0.1822**	**40.5**	**–19.6**	**263.5**	**53.98**
20.56 (–*x*)	1.3128	0.1805	41.1	–22.5	280.1	63.18
	**1.3128**	**0.1932**	**42.8**	**–18.2**	**262.8**	**50.39**
25.70 (–*x*)	1.3069	0.1882	45.2	–21.1	272.5	60.26
	**1.3032**	**0.1968**	**41.5**	**–17.6**	**259.1**	**51.28**
30.84 (–*x*)	1.2982	0.1913	40.6	–20.3	270.6	57.27
	**1.2918**	**0.2032**	**42.6**	**–16.9**	**257.3**	**48.87**
35.97 (–*x*)	1.2806	0.1989	43.5	–19.2	268.1	54.32
	**1.2869**	**0.2069**	**43.7**	**–16.2**	**255.8**	**47.69**
41.12 (–*x*)	1.2733	0.2015	41.8	–18.8	265.3	52.28
	**1.2758**	**0.2113**	**45.1**	**–15.6**	**252.9**	**43.13**
	1.2682 [a]	*0.2105*	*45.2*	*–15.6*	*254.8*	*55.16*
	1.2642 [a]	***0.2182***	***43.8***	***–13.8***	***255.3***	***41.10***
41.12 (+*y*)	1.3728	0.1392	42.7	–19.7	268.9	52.17
	**1.3850**	**0.1358**	**42.8**	**–17.2**	**247.1**	**40.88**
35.97 (+*y*)	1.3692	0.1417	43.5	–19.7	270.6	57.62
	**1.3765**	**0.1440**	**44.0**	**–18.2**	**248.3**	**45.39**
30.84 (+*y*)	1.3628	0.1425	44.2	–20.7	272.5	59.38
	**1.3673**	**0.1478**	**45.1**	**–18.8**	**251.2**	**48.35**
25.70 (+*y*)	1.3589	0.1438	43.2	–21.7	274.6	60.29
	**1.3621**	**0.1522**	**42.5**	**–19.2**	**252.3**	**50.62**
20.56 (+*y*)	1.3536	0.1501	41.5	–22.3	282.3	62.18
	**1.3589**	**0.1563**	**40.9**	**–19.6**	**258.7**	**52.24**
15.42 (+*y*)	1.3501	0.1572	41.5	–23.8	286.9	65.76
	**1.3518**	**0.1628**	**39.3**	**–20.5**	**262.1**	**54.18**
10.28 (+*y*)	1.3467	0.1583	38.5	–24.3	292.8	67.37
	**1.3455**	**0.1659**	**39.2**	**–20.7**	**266.5**	**55.62**
5.14 (+*y*)	1.3428	0.1602	40.5	–25.0	294.1	68.13
	**1.3421**	**0.1683**	**34.3**	**–17.9**	**267.2**	**57.23**
5.14 (–*y*)	1.3401	0.1626	42.1	–25.6	294.5	70.18
	**1.3385**	**0.1736**	**37.5**	**–21.2**	**271.3**	**57.27**
10.28 (–*y*)	1.3362	0.1659	41.6	–24.8	296.2	72.26
	**1.3321**	**0.1767**	**37.1**	**–20.5**	**273.5**	**59.31**
15.42 (–*y*)	1.3276	0.1679	41.1	–26.2	299.8	73.59
	**1.3265**	**0.1788**	**36.2**	**–22.8**	**276.8**	**56.62**

20.56 (–*y*)	1.3218	0.1685	40.5	–25.3	302.3	75.38
	**1.3201**	**0.1795**	**35.8**	**–24.0**	**280.2**	**53.53**
25.70 (–*y*)	1.3155	0.1718	39.8	–26.8	306.8	72.29
	**1.3188**	**0.1805**	**35.2**	**–23.8**	**283.5**	**56.18**
30.84 (–*y*)	1.3082	0.1723	38.2	–24.6	310.9	71.82
	**1.3132**	**0.1836**	**34.6**	**–21.1**	**286.7**	**57.26**
35.97 (–*y*)	1.2988	0.1757	37.6	–23.2	313.6	73.63
	**1.3046**	**0.1862**	**33.8**	**–20.7**	**289.8**	**58.32**
41.12 (–*y*)	1.2815	0.1796	36.1	–25.1	316.7	77.69
	**1.2969**	**0.1885**	**32.1**	**–20.5**	**291.3**	**53.17**

[a] In aqueous solution.

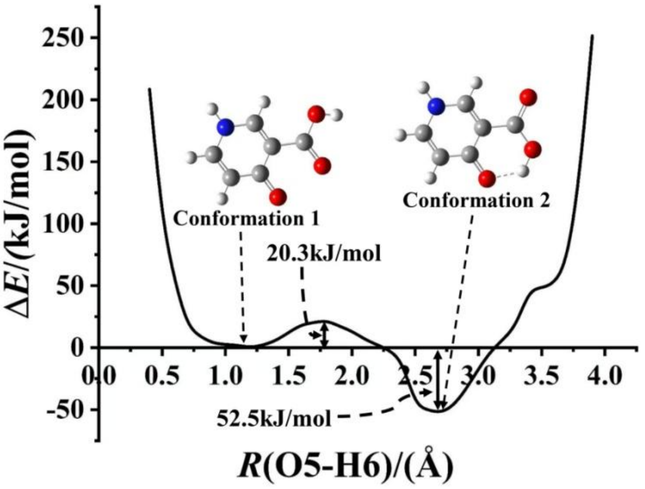

Figure S1 Scheme of energy barriers on proton transfer and transformation for 4-pyridone-3-carboxylic.

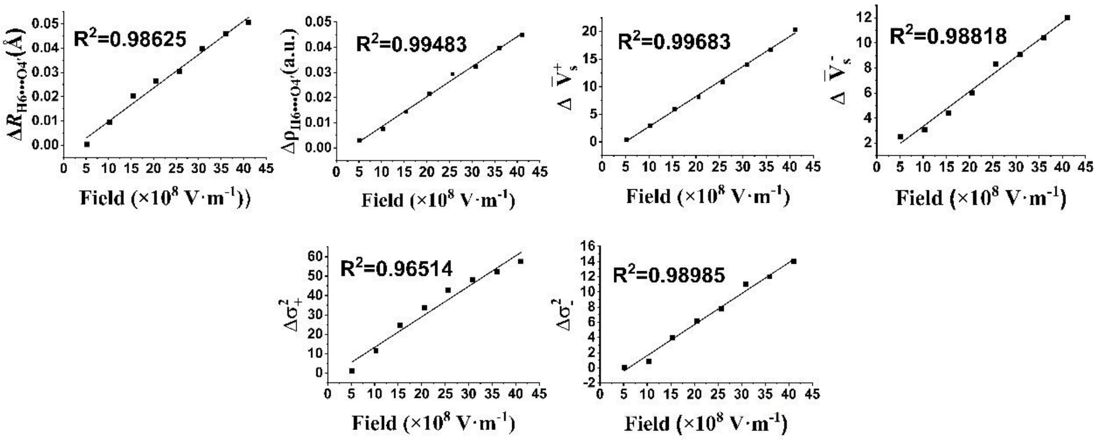

(a) M06-2X/6-311++G (+*x*)**

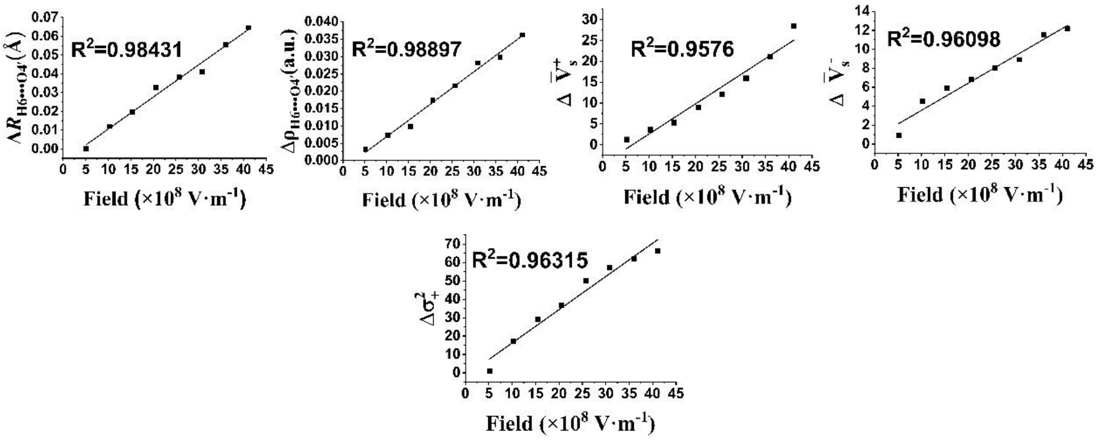

(b) M06-2X/aug-cc-pVTZ (+*x*)

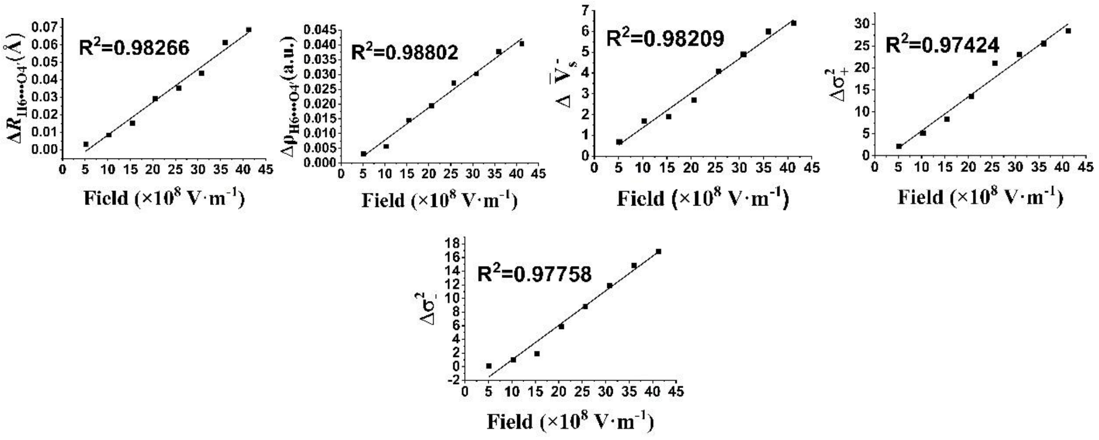

(c) M06-2X/6-311++G (–*x*)**

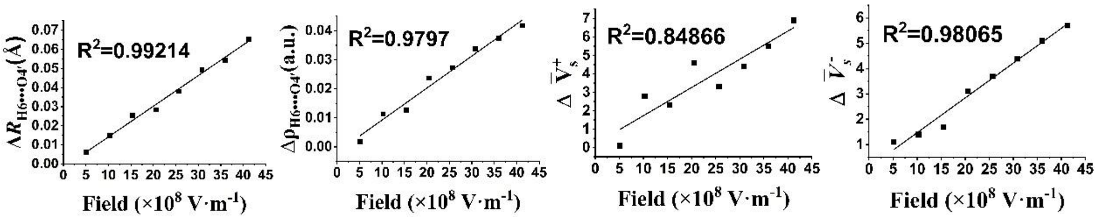

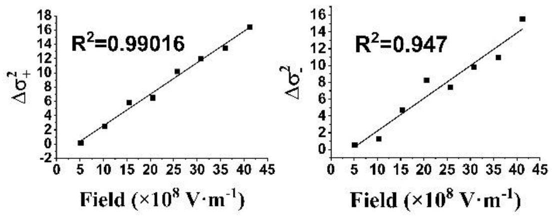

(d) M06-2X/aug-cc-pVTZ (–*x*)

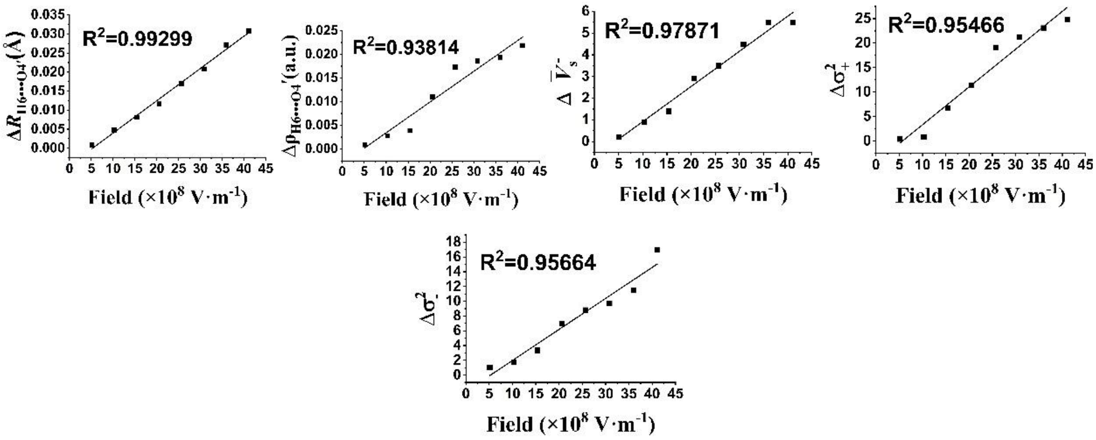

(e) M06-2X/6-311++G (+*y*)**

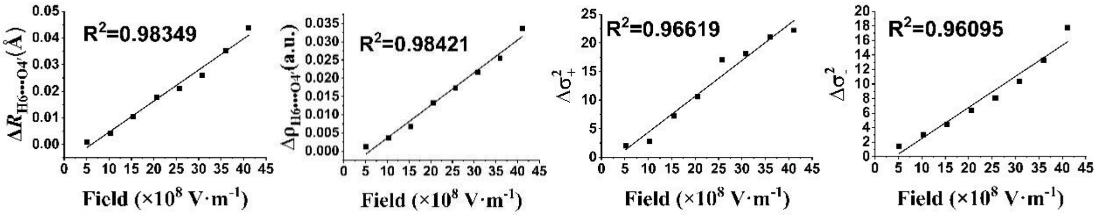

(f) M06-2X/aug-cc-pVTZ (+*y*)

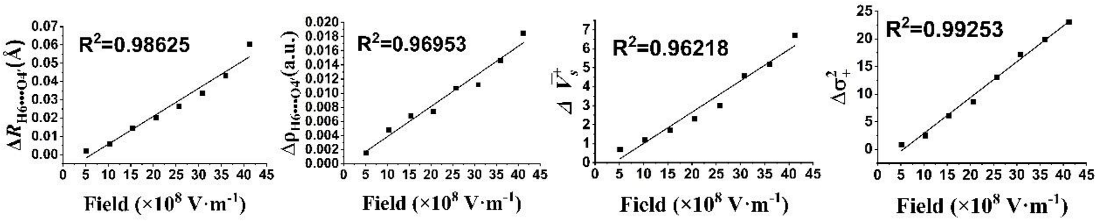

(g) M06-2X/6-311++G (–*y*)**

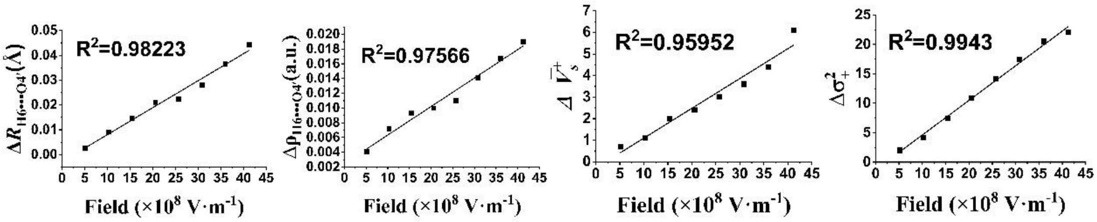

(h) M06-2X/aug-cc-pVTZ (–*y*)

Figure S2 Relationships between the structures, AIM, electrostatic potential results of TS and external electric field strengths at the M06-2X/6-311++G** and M06-2X/aug-cc-pVTZ levels with different electric field directions (+*x*-, –*x*-, +*y*-,and –*y*-directions).

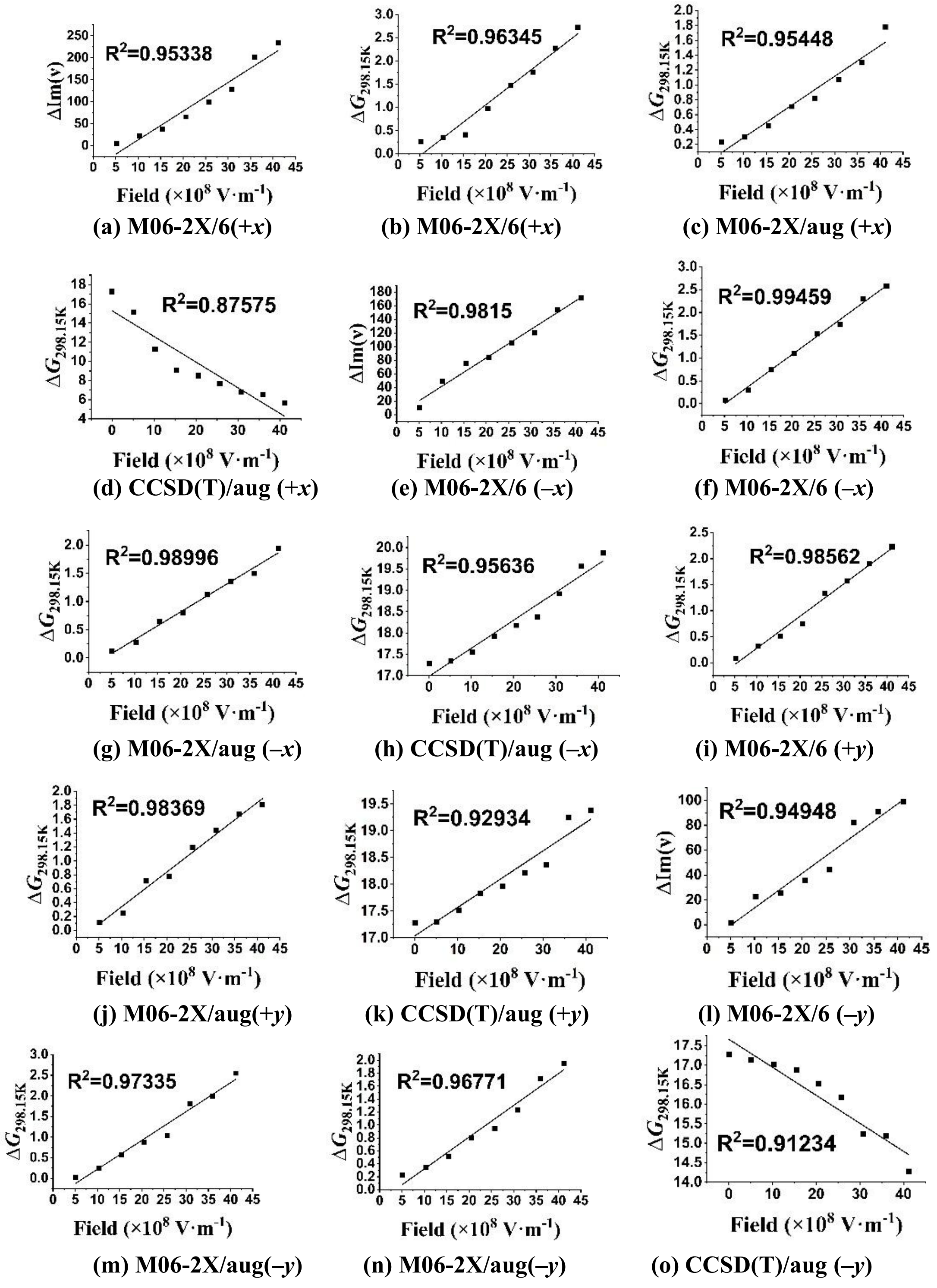

Figure S3 Selected relationships between the frequencies, barrier heights (ΔG) and external electric field strengths at the M06-2X/6-311++G** (i.e., M06-2X/6), M06-2X/aug-cc-pVTZ (i.e., M06-2X/aug) and CCSD(T)/aug-cc-pVTZ (i.e., CCSD(T)/aug) levels with different electric field directions (+*x*-, –*x*-, +*y*-,and –*y*-directions).
